# Peroral Endoscopic Myotomy for Achalasia after Bariatric Surgery: A Case Report and Review of the Literature

**DOI:** 10.3390/diagnostics13213311

**Published:** 2023-10-26

**Authors:** Roberta Maselli, Matteo Fiacca, Gaia Pellegatta, Roberto de Sire, Federico De Blasio, Antonio Capogreco, Piera Alessia Galtieri, Davide Massimi, Manuela Trotta, Cesare Hassan, Alessandro Repici

**Affiliations:** 1 Humanitas Clinical and Research Center—IRCCS, 20089 Milan, Italycesare.hassan@hunimed.eu (C.H.); alessandro.repici@hunimed.eu (A.R.); 2 Department of Biomedical Sciences, Humanitas University, 20090 Milan, Italy; 3 Department of Surgery, Oncology and Gastroenterology, University of Padua, 35128 Padua, Italy; 4 Gastroenterology, Department of Clinical Medicine and Surgery, University Federico II, 80138 Naples, Italy; 5 Clinic of Gastroenterology, Hepatology and Emergency Digestive Endoscopy, University Politecnica delle Marche, 60126 Ancona, Italy

**Keywords:** bariatric surgery, achalasia, dysphagia, POEM

## Abstract

Introduction: Achalasia following bariatric surgery is a rare phenomenon with diverse potential physiopathological origins. Aims: This article aims to explore the hypothetical physiopathological connection between bariatric surgery and the subsequent onset of achalasia. Material and Methods: A review was conducted to identify studies reporting cases of peroral endoscopic myotomy (POEM) after bariatric procedures and detailing the outcomes in terms of the technical and clinical success. Additionally, a case of a successful POEM performed on a patient two years after undergoing laparoscopic sleeve gastrectomy (LSG) is presented. Results: The selection criteria yielded eight studies encompassing 40 patients treated with POEM for achalasia after bariatric surgery: 34 after Roux-en-Y gastric bypass (RYGB) and 6 after LSG. The studies reported an overall technical success rate of 97.5%, with clinical success achieved in 85% of cases. Adverse events were minimal, with only one case of esophageal leak treated endoscopically. However, a postprocedural symptomatic evaluation was notably lacking in most of the included studies. Conclusions: Achalasia poses a considerable challenge within the bariatric surgery population. POEM has emerged as a technically viable and safe intervention for this patient demographic, providing an effective treatment option where surgical alternatives for achalasia are limited. Our findings highlight the promising outcomes of POEM in these patients, but the existing data remain limited. Hence, prospective studies are needed to elucidate the optimal pre-surgical assessment and timing of endoscopic procedures for optimizing outcomes.

## 1. Background

Achalasia is a rare chronic disorder of the esophageal smooth muscle characterized by impaired relaxation of the lower esophageal sphincter (LES) and absent or spastic contractions in the esophageal body. The key pathophysiological mechanism is the loss of inhibitory nerve function that probably results from an autoimmune attack targeting the esophageal myenteric nerves through cell-mediated and, possibly, antibody-mediated mechanisms. Epidemiologic studies have underlined that morbidly obese patients are more prone to developing esophageal motility disorders such as achalasia, compared with the general population, with approximately 50% of obese patients in one prospective cohort study demonstrating dysmotility on the esophageal manometry [1,2,3].

Moreover, several reports have described achalasia in patients who undergo bariatric surgery, hypothesizing that it could be due to the mechanical effect of gastric banding, neurological damage during the surgical procedure, or an endocrinological alteration due to modified gastric secretions [4,5,6]. Traditional therapies for achalasia include pneumatic dilation, endoscopic injection of botulinum toxin, and laparoscopic Heller myotomy (LHM). POEM has more recently emerged as a safe and effective treatment modality for achalasia and can, furthermore, be successfully performed in patients who have had prior endoscopic or surgical interventions [7,8,9]. Randomized trials and meta-analyses have shown that its efficacy is superior to that of pneumatic dilation and comparable to the traditional gold-standard surgical approach represented by LHM, with an outstanding safety profile [10,11,12]; higher post-operative reflux rates seem to be associated with POEM over LHM [13]. POEM has consequently been proposed as a first-line option over LHM for patients with previous abdominal surgery or obesity, which may lead to higher peri- and post-operative risk [14]. Although most of the data accumulated have been on treatment-naïve patients or patients with a previous pneumatic dilation, over the last years, POEM has also been successfully performed in patients with previous LHM, confirming its feasibility even in a surgically altered gastroesophageal junction (GEJ). 

However, some concerns have been raised about the technical feasibility of POEM in post-bariatric patients: surgical sutures may be found in the dissection plane (especially on the gastric side after LSG) [15]. RYGB and LSG are both procedures that may cause an adherence formation near the GEJ, making submucosal tunneling challenging, and, moreover, the reduced gastric lumen may hamper tunneling into the gastric side. 

The number of people undergoing bariatric procedures is constantly increasing, so the rates of patients that may develop achalasia and be subsequently treated with POEM is thought to increase as well. Data are, however, scarce and mostly limited to case reports or small series, some of which obtained conflicting results. 

Given the two different cases of POEM performed in our unit after bariatric surgery [16] and to address the need for further knowledge about the efficacy and safety of a POEM after bariatric surgery, we aimed to review the available literature on the subject. 

## 2. Materials and Methods

A literature review was performed to identify studies reporting on POEM after bariatric procedures. The search was conducted on PUBMED/Medline, EMBASE and Scopus databases using a combination of the following medical terms: “achalasia”, “POEM”, “endoscopic myotomy”, “bariatric”, “sleeve” and “gastric bypass”. All studies published from inception to July 2023 were screened by title and abstracts and considered for eligibility. References of review articles were also hand-searched. Eligibility criteria included endoscopic treatment of achalasia after bariatric surgery and reporting on outcomes of technical and clinical success after POEM. Exclusion criteria were POEM performed for non-achalasia motility disorder, co-performance of POEM and bariatric procedure, surgical treatment and review or editorial articles. In case of any suspicion of cohort overlap, the most recent study was included.

Data regarding demographic, clinical and procedural variables (sex, age, bariatric intervention, time of achalasia development after surgery, manometric subtype) were extracted from all studies included and reported in a standardized Excel database. Endpoints of interest were technical success, clinical success, adverse events, postprocedural reflux and PPI use. Statistical analysis was limited to descriptive analysis (frequencies and percentages; mean, median and standard deviations and ranges). Pooled analysis was not performed due to the paucity of the population included.

Additionally, a case of a successful POEM performed in our Institute on a female patient diagnosed with achalasia 2 years after LSG is presented. 

## 3. Case Report

**Figure 1:**
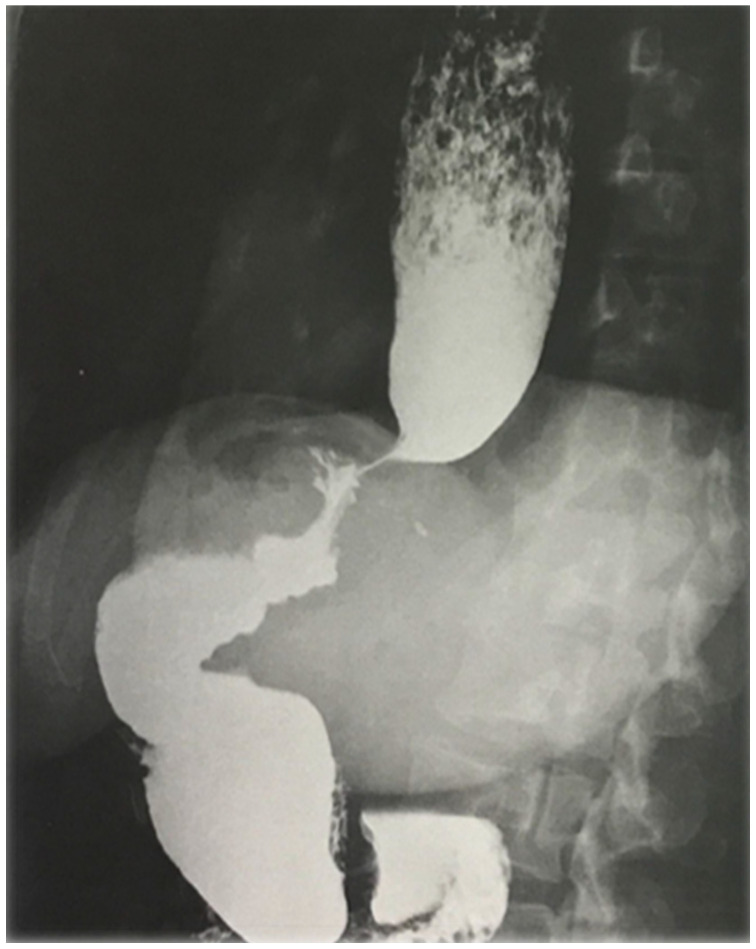
Barium esophageal X-ray showing marked esophageal dilation and contrast stasis suggestive of achalasia. Gastric sleeve appears regular.

**Figure 2:**
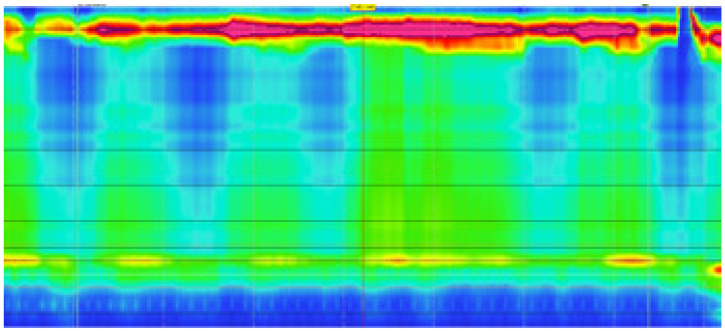
High-resolution manometry showing increased IRP with failed swallow-induced peristalsis and pan-esophageal pressurization; suggestive of type II Achalasia diagnosis.

**Figure 3:**
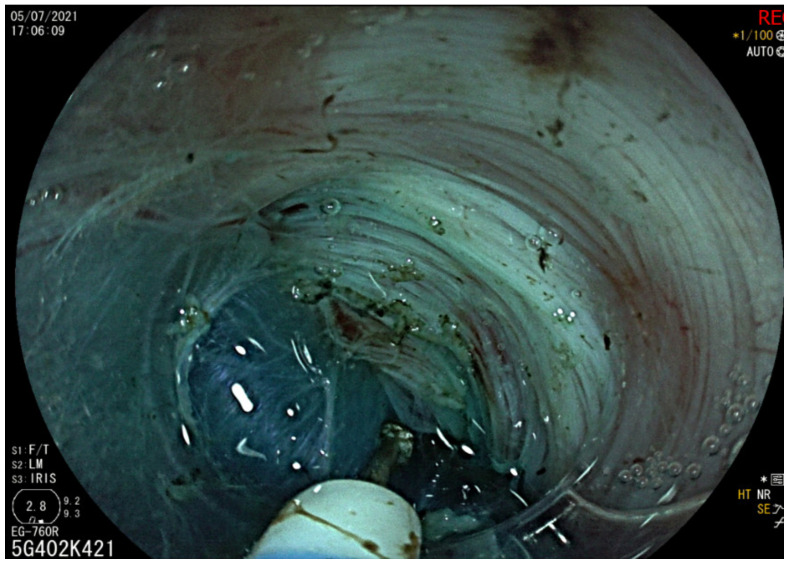
POEM: Submucosal tunneling step.

**Figure 4:**
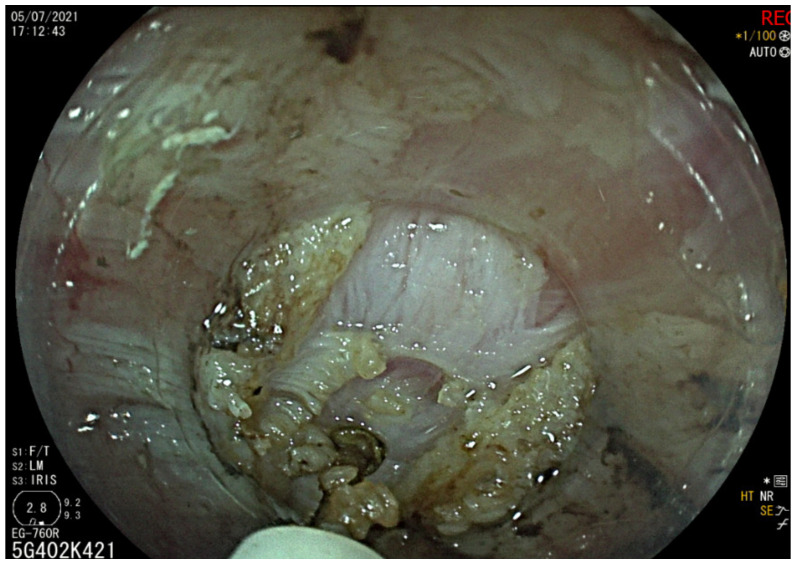
POEM: Myotomy phase of the procedure.

A 42-year-old woman was referred by the bariatric surgeons to our gastroenterology clinic for dysphagia, regurgitation, chest pain and weight loss of 10 kg (BMI 24.1), with an Eckardt score of 9. She had undergone LSG 26 months earlier (BMI 41.4), and symptoms started nearly 1 year after surgery, with dysphagia worsening after a successful childbirth. A timed barium esophageal X-ray was initially performed, showing a dilated esophagus with esophageal contrast stasis. No issues related to the previous bariatric surgery were found (Figure 1). The upper endoscopy confirmed esophageal dilation with food stasis, while the gastric sleeve was regular. High-resolution manometry confirmed a type II achalasia with an LES-integrated relaxation pressure (IRP) of 39.3 mmHg (Figure 2). POEM was planned and performed according to our protocol (Figure 3 and Figure 4). Technical success was achieved, and anterior myotomy was performed without issues, although distal tunneling inside the gastric side was technically difficult because of increased submucosal fibrosis, likely related to postsurgical alterations. No adverse procedural events occurred. The post-operative course was uneventful, a soft oral diet was started after 24 h, and the patient was discharged the day after the procedure. At her first follow-up visit after three months from the procedure, the patient underwent upper gastrointestinal endoscopy and pH-metry, which showed grade A esophagitis with a normal acid exposure time: 0.8% (Demeester Score: 2). The high-resolution manometry showed a normal LES-IRP after POEM (7.4 mmHg). The patient reported a complete resolution of dysphagia and chest pain (Eckardt score of 0) with a weight regain of 4 kg and only complained of mild reflux symptoms, which were controlled with mild proton-pump inhibitor therapy (omeprazole 20 mg). The patient has currently undergone 20 months of follow-up, with no recurrence of dysphagia or reflux-induced symptoms.

## 4. Results

A total of nine articles, including a total of 40 patients who developed achalasia after bariatric surgery resulted from the literature research. One article was excluded because follow-up data were not reported. Eight articles were included in the final analysis. The studies were published between 2014 and 2023. Seven studies were performed in the United States, with only one European report; four studies were single-patient case reports. Six studies were single-center-based, two were multi-centric; all studies had a retrospective design. Studies and patient characteristics are summarized in Table 1. 

Two studies (Bashir et al. [20] and Craft et al. [21]) included patients with post-bariatric-surgery achalasia treated with different procedures. In these cases, only POEM cases were evaluated in these series [20,21]. Moreover, Bashir et al. included a total of six patients with achalasia prior to and following RYGB, but two of these had achalasia prior to RYGB and were not evaluated in our review [20]. In the Craft et al. series, 9 of 13 patients were diagnosed with achalasia after bariatric surgery and of these, only one was treated with POEM [21]. 

The eight articles included the reported outcomes of 40 patients treated with POEM for achalasia after bariatric surgery (34 RYGB, 6 LSG) [15,17,18,20,21,22,23]. In the case reported by Donatelli et al., POEM was performed in a female patient with prior multiple bariatric surgical procedures: a laparoscopic lap band, followed by LSG and, finally, by RYGB [22]. In the total population, 9/39 (23%) were men (one study did not specify the sex of the patient included); the mean age was 54.8 years (±8.85 years). The mean time from bariatric surgery to POEM was 8.86 ± 2.66 years (reported in eight studies). The mean follow-up time was 35.5 ± 27.5 months. According to the current Chicago classification, 12 patients (30%) had type I, 19 patients (47.5%) had type II and 9 patients (22.5%) had type III manometric sub-type of achalasia. The pre-POEM mean IRP was 25.4 ± 1.2 mm Hg (four studies did not specify the value), whereas the post-POEM IRP value was missing in all the studies reported. The pre-POEM mean Eckardt score was 6.91 ± 1.79 and the post-POEM Eckardt score was 1.6 ± 1.4 (two studies did not specify the data). Twenty patients (50%) had undergone previous therapies for achalasia, namely, three (7.5%) had undergone a prior Heller myotomy, nine (22.5%) had undergone a botulinum toxin injection and nine (22.5%) patients had undergone pneumatic dilation (in the Kolb et al. series, one patient had undergone both botulinum toxin injection and Heller myotomy). Overall, there were 20 (50%) naïve patients, with no previous treatment for achalasia. Technical success was achieved in 39 cases (97.5%), and the only technical failure was reported in the study by Bomman et al. [23], where one patient had an esophageal leak that was managed endoscopically. Clinical success was reported in 34 patients (85%). Recurrence of symptoms was reported in one patient with type III achalasia in the series by Bashir et al. [20]; the only patient evaluated in the study by Crafts et al. [21] underwent pneumatic dilatation after POEM, and should therefore be considered a clinical failure of POEM; in the study by Kolb et al. [15], out of six patients, the authors reported that one had recurrent candida esophagitis, whereas another had relapsing achalasia symptoms. Despite Bomman et al. [23] declaring a clinical success rate of 93.8% [15,16], with one patient undergoing pneumatic dilation after POEM, symptoms recurred in two patients (3 months and 12 months after POEM), and they were both considered a clinical failure in our analysis. In all the studies, there were no severe adverse events reported, with the only one being one esophageal leak [23], which also resulted in a technical failure. A postprocedural symptomatic evaluation was lacking in almost all of the studies included in this review. The study by Kolb et al. specified that two out of the three patients with prior LSG had LA grade A/B reflux esophagitis, while in the third patient, candida esophagitis was described. In the study by Bomman et al., 5/16 patients complained of reflux symptoms after POEM [15]. None of the included studies reported if patients were placed on PPI therapy after POEM.

## 5. Discussion 

### 5.1. Achalasia Developing after Bariatric Surgery: A Link to the Pathogenesis?

The incidence of obesity in the general population is increasing, and according to recent estimates, the prevalence in the US may be near 40% of the adult population [24]. The number of patients with morbid obesity who undergo bariatric surgery is therefore set to steadily increase over the next decades, having already doubled over the last 10 years [24]. 

Esophageal motor disorders are not common in the general population, although an increasing trend is reported: recent epidemiological studies have shown that the prevalence of achalasia in the general population may be around 3 per 10,000, with an incidence near 3 per 100,000 per year [25,26]. These figures nearly double previous incidence estimates [27]. Whether this increase is related to the widespread diffusion of high-resolution manometry, improved case collection, or an actually increasing incidence is still under debate. 

There is a robust body of evidence that suggests that the incidence of such disorders may be higher in obese patients [3]. Several factors may contribute to this process. Mechanically, an increased abdominal pressure may represent an obstacle to bolus progression into the abdominal cavity; moreover, it can facilitate gastroesophageal reflux, whose incidence is known to be higher in obese patients. Gastroesophageal reflux is a known risk factor for esophageal dysmotility, with cases directly progressing to achalasia also described [28]. Leptins have been hypothesized to play a role as a hormonal factor. Leptin is a hormone derived from adipose tissue that acts by modulating appetite and energy control. Its plasmatic concentration is thought to be altered in obese patients, tending to higher levels [29]. Studies have shown that it may decrease gastric and intestinal motility [30]. Schrumpf et al. hypothesized a possible hormonal effect caused by the postprandial gastrin decrease with a consequent significant rise in the LES resting pressure [5].

Peristalsis and LES opening are two esophageal physiologically crucial steps that are both mediated by vagal fibers, so it is logical to believe that the dysfunction of one of them may play a role in esophageal motility disorders. In the past century, surgical vagotomy has been examined as a model for achalasia in animals: an achalasia-like syndrome was obtained in dogs after electrolytic lesions of the medulla and bilateral vagotomy [31]. Manometric and histologic findings were, however, different from those observed in achalasia. Another investigation of the effects of cervical vagotomy in primates showed that only two of seven monkeys developed a radiographic and manometric presentation consistent with achalasia [32]. Interestingly, the majority of animals demonstrated a reduction in the number of esophageal myenteric ganglion cells. Transection or cooling of the vagus nerve has also been shown to abolish primary peristalsis but leave secondary peristaltic function intact in the opossum [33,34].

A neuropathic dysfunction due to direct iatrogenic vagal nerve damage or surgical trauma has been suggested as a pathogenic factor of achalasia by some authors, with consequent development of new or worsening of previous asymptomatic motility disorders [35,36]. Data have been published on achalasia development after gastroesophageal anti-reflux surgery, mostly fundoplications, although most publications are single-case reports, so the paucity of observations cannot assure the replicability of these findings [37,38]. It is a recent observation that people undergoing bariatric surgery may have a higher likelihood of developing esophageal motor disorders. According to a recent study, over a median of 5.84 years after bariatric surgery, out of 97 patients who underwent esophageal high-resolution manometry (indication in nearly 50% was dysphagia), achalasia was found in 7 (7.2%) [39]; in the control group of 40 pre-bariatric patients, no cases of achalasia were found. This is a remarkable finding, even considering that selection bias may have occurred: when correcting the analysis based on the average observed annual bariatric surgery volume (640 patients), the estimated incidence of post-bariatric surgery achalasia would be around 0.16%, nearly 100 times that of the general population. 

Pseudo-achalasia occurring secondarily to gastric banding placement has been extensively reported, with a rather logical explanation that the banding placement acts as a factual gastroesophageal obstruction [40]. More recently, an increasing number of achalasia cases have been described after RYGB [36,41,42]. The first pathogenic mechanism underlying achalasia development may be the direct disruption of vagal fibers. Although surgical maneuvers when performing RYGB or LSG should not involve the gastroesophageal junction, inadvertent transection of vagal branches may still occur, in particular if a hiatal hernia is present and reduced. Another mechanism sees intra-abdominal pressure playing the trigger role: it is known that in non-operated obese patients, increased abdominal pressure may facilitate gastroesophageal reflux, as well as hamper bolus progression into the abdominal cavity. A possibly stronger obstruction may result after lumen-restricting bariatric procedures such as RYGB or LSG. In this setting, the presence of a functional gastroesophageal outflow obstruction, if accompanied by an ineffective body motility, may explain the development of achalasia. A recent paper by Miller et al. provides a fresh interpretation of esophageal dysfunction after bariatric surgery, which sees the functional gastroesophageal outflow obstruction playing a crucial role [39]. It has been postulated that a unique achalasia-like pattern may develop after the bariatric anatomic alteration, which the authors termed post-obesity surgery esophageal dysfunction (POSED). POSED was defined by the coexistence of normal IRP, elevated intragastric pressure (defined as 30 mmHg when not accompanied by a simultaneous elevation in intrathoracic pressure), and complete aperistalsis of the esophagus. In the study, this unique manometric abnormality was found in as much as 5.2% of the population. 

Previous studies have suggested that the loss of esophageal body function in achalasia may be a result (secondary aperistalsis) of the gastroesophageal outflow obstruction of a nonrelaxing lower esophageal sphincter [43]. In line with this observation, our novel hypothesis is that the distal functional obstruction typical of POSED may represent the initial trigger of a secondary aperistalsis, which eventually leads to the progressive neuromuscular degeneration that is typical of primary achalasia. 

### 5.2. POEM in Post-Bariatric Patients 

POEM is a promising treatment modality for the management of patients with achalasia. Recent studies have reported excellent short-term outcomes in patients with achalasia who were treated with POEM, although data regarding the long-term outcomes for esophageal motility disorders in post-bariatric patients are limited. This is a technical challenge for most endoscopists, even in tertiary centers for third-space endoscopy. There is still little and weak evidence given the small number of cases of such a rare disorder and the growing experience in this field. International data and clinical practice have evidenced that the POEM procedure can be performed with optimal technical and clinical success rates despite initial concerns and debates on its feasibility. 

Altered anatomies and GEJ function, fibrosis and a lack of gastric space could interfere with the creation of submucosal tunneling and subsequent myotomy, making the endoscopic procedure more difficult and insidious than the ordinary [36]. 

Surgery, on the contrary, is hampered by a high risk of complication and technical failure because of adhesions and anatomy alterations. It must also be underlined that the advantage of combining treatment with an anti-reflux procedure during LHM is lost, since fundoplication is not feasible after bariatric surgery [44,45]. In the studies we analyzed, the POEM procedure in patients with previous bariatric surgery was not deemed technically more challenging than that performed in patients without prior RYGB or LSG, and, in agreement with previous reports, it seems to be feasible, effective and safe in patients with prior bariatric surgery [15,19,23]. Our group recently reported the first outcomes of POEM for achalasia after a vertical banded gastroplasty, which showed technical and clinical success with single-dose PPI-controlled gastro-esophageal reflux disease (GERD) at the following visits [16]. In this review, we report the other case performed at our institute. 

Given the technical and clinical drawbacks associated with surgery and the promising evidence in favor of POEM, the endoscopic approach can become the procedure of choice for these patients. The principal limitations of the current literature and of our narrative review are the small number of reports and the possible background selection bias related to the heterogeneity of the included studies and methodological quality. Patients were treated in different centers with diverse expertise and endoscopic/surgical skills. The lack of follow-up data in addition to the paucity of manometric patterns, pH study data, and Eckardt score evaluations are other limitations, especially regarding postprocedural GERD findings. Post-POEM GERD has been reported in 31%–58% of cases in general conditions, with a slight decrease over time and with a good response to oral treatment with PPI [46,47]. Post-bariatric patients may be at a higher risk of GERD and its complications given the more significant GEJ alterations. In our review, clinical data, based on patients’ symptoms of reflux, seem to resemble standard post-POEM experiences, but further data are necessary in order to draw a conclusion. 

Moreover, because of the rarity of this condition, it is challenging to perform a large prospective study to compare surgical and endoscopic procedures. It is likely that because of the increasing number of morbidly obese patients and concomitant worldwide growth in weight-loss procedures, the number of these patients will increase in the future.

## 6. Conclusions 

POEM appears to be safe and effective in treating patients with achalasia who have undergone prior bariatric surgery (RYGB and LSG). With recent emerging evidence of the increased prevalence of achalasia and other major esophageal dysmotility disorders as a time-dependent complication of bariatric surgery, POEM may be a suitable treatment option for these patients in scenarios where surgical options are limited. 

Moreover, further clinical pre-bariatric evaluations should be considered in order to identify pre-existing esophageal motility disorders early in obese patients. Available data are promising but still poor; therefore, prospective studies with larger sample sizes, homogeneous variables such as technical and clinical success, postprocedural recurrence and GERD with a longer follow-up are needed to confirm existing evidence.

## Figures and Tables

**Table 1:** Study characteristics. POEM: peroral endoscopic myotomy; Pts: patients; BMI: body mass index; F.U.: follow-up; AEs: adverse events; RYGB: roux-en-y gastric bypass; ORYBG: open roux-en-y gastric bypass; LRYGB: laparoscopic roux-en-y gastric bypass; LGB: laparoscopic gastric banding; SG: sleeve gastrectomy.

Study	Year	Country	Design	Bariatric Procedures	Interval from Surgery to POEM (Years)	Pts	Mean Age [SD]	BMI [SD]	Achalasia Type			Mean Baseline IRP (mmHG) [SD]	Mean Baseline Eckardt [SD]	Mean Post-POEM Eckardt [SD]	Mean F.U. (Weeks) [SD]	Severe AEs
									I	II	III					
Yang and Draganov [17]	2014	USA	Monocentric	RYGB	\	1	64	\	1	0	0	\	4	0	5	0/1
Luo et al. [18]	2017	USA	Monocentric	RYGB	12	1	67	24.94	0	0	1	26.6	\	\	24	0/1
Sanaei et al. [19]	2019	USA, Denmark	Multicentric	3 ORYGB, 7 LRYGB	7.5	10	52.5 [13.4]	\	5	4	1	25 [9.7]	6.5	1	19.3	\
Bashir et al. [20]	2019	USA	Monocentric	RYGB	6	4	42 [18.4]	33.4 [8.7]	0	2	2		8.2 [1.5]	1.75 [3.5]	34 [16.5]	0/4
Crafts et al. [21]	2020	USA	Monocentric	RYGB	13	1	\	\	1	0	0	\	\	\	48	0/4
Kolb et al. [15]	2020	USA	Monocentric	3 SG, 3 RYGB	7	6	47.6 [13.3]	\	2	4	0	23.8 [3.1]	7.6 [2.4]	4.1 [3.2]	84	0/6
Donatelli et al. [22]	2021	France	Monocentric	LGB+ SG+ LRYGB	9	1	58	22	1	0	0	\	9	1	8	0/1
Bomman et al. [23]	2021	USA	Multicentric	2 SG, 14 RYGB	7.5	16	52.3 [16.7]	33.6 [7.6]	2	9	5	26.2 [7.6]	6.1 [2.1]	1.7 [1.8]	62	1/16

## Data Availability

Not applicable.
